# Protective effect of carvedilol alone and coadministered with diltiazem and prednisolone on doxorubicin and 5‐fluorouracil‐induced hepatotoxicity and nephrotoxicity in rats

**DOI:** 10.1002/prp2.381

**Published:** 2018-01-12

**Authors:** Abidemi J. Akindele, Gabriel O. Oludadepo, Kennedy I. Amagon, Dhirendra Singh, Daniel D. Osiagwu

**Affiliations:** ^1^ Department of Pharmacology Therapeutics and Toxicology Faculty of Basic Medical Sciences College of Medicine University of Lagos Lagos Nigeria; ^2^ Department of Pharmacology Faculty of Pharmaceutical Sciences University of Jos Jos Nigeria; ^3^ Department of Pharmacology Shakambhari Institute of Higher Education and Technology Roorkee India; ^4^ Department of Anatomic and Molecular Pathology Faculty of Basic Medical Sciences College of Medicine University of Lagos Lagos Nigeria

**Keywords:** antineoplastic toxicity, antioxidant, carvedilol, diltiazem, prednisolone

## Abstract

This study investigated the protective effects of carvedilol alone and coadministered with prednisolone and diltiazem on doxorubicin (DOX) and 5‐fluorouracil (5‐FU)‐induced toxicity. Each of 2 pools of 70 female rats were randomly allotted into 10 groups of 7 animals each and treated as follows: Group 1: normal saline (10 mL/kg); Group 2: normal saline and DOX (40 mg/kg)/5‐FU (20 mg/kg) alone; Group 3: gallic acid (200 mg/kg) and DOX/5‐FU; Group 4: carvedilol (0.075 mg/kg) and DOX/5‐FU; Group 5: carvedilol (0.15 mg/kg) and DOX/5‐FU; Group 6: carvedilol (0.30 mg/kg) and DOX/5‐FU; Group 7: diltiazem (3.43 mg/kg) and DOX/5‐FU; Group 8: diltiazem (3.43 mg/kg), carvedilol (0.15 mg/kg), and DOX/5‐FU; Group 9: prednisolone (0.57 mg/kg) and DOX/5‐FU; and Group 10: prednisolone (0.57 mg/kg), carvedilol (0.15 mg/kg), and DOX/5‐FU. Treatments were done p.o. for 16/14 days for the DOX/5‐FU models. DOX/5‐FU was administered i.p. to the rats in Groups 2‐10 on day 14/10‐14. On day 17/15 (DOX/5‐FU), blood samples were collected, and liver and kidneys of rats were harvested for antioxidant and histopathological assessments. Carvedilol alone and coadministered with prednisolone significantly (*P* < .05) decreased alanine aminotransferase level compared with administration of DOX alone. Carvedilol alone and coadministered with diltiazem significantly (*P* < .05) decreased creatinine level compared with administration of DOX/5‐FU alone. Carvedilol alone and coadministered with diltiazem and prednisolone significantly (*P* < .05) increased the level of hepatic superoxide dismutase and catalase, and decreased malondialdehyde compared with DOX administration alone. Histopathological observations correlated with results of biochemical and antioxidant analyses. Carvedilol administered alone and coadministered with diltiazem and prednisolone reduced the effect of DOX/5‐FU‐induced hepatic and renal toxicities due to enhanced in vivo antioxidant activity. The protective effect was more prominent in the doxorubicin model compared with the 5‐fluorouracil test. Coadministration of carvedilol with either diltiazem or prednisolone did not show better protection relative to carvedilol alone.

**Abbreviation**
ALP: alkaline phosphatase
ALT: alanine aminotransferase
AST: aspartate aminotransferase
CAT: catalase
FU: 5‐fluorouracil
GPx: glutathione peroxidase
GSH: reduced glutathione
HDL: high‐density lipoprotein
LDL: low‐density lipoprotein
MDA: malondialdehyde
NADH‐D: nicotinamide adenine dinucleotide dehydrogenase
SOD: superoxide dismutase
TG: triglycerides

## 1 INTRODUCTION

Chemotherapy remains a strong indication for the treatment of cancer patients and antineoplastic agents like doxorubicin, 5‐fluorouracil, and cisplatin are often used in the treatment of cancers. The major problems associated with antineoplastic agents include resistance, secondary malignancy, cost, and lack of selectiveness resulting in negative impact on normal cells of the body. Doxorubicin is used to treat different forms of cancer, including ovarian, breast, lung, uterine and cervical cancers, Hodgkin's disease, and soft tissue and primary bone sarcomas.^1^ The use of this drug is limited by toxic effects on body organs, causing cardiac, pulmonary, hepatic, renal, hematological, and testicular toxicities.^2^ 5‐Fluorouracil (5‐FU), an antimetabolite, has played an important role in the management of colon and breast cancers, and cancers involving the head and neck.^3^ Despite the many advantages, its clinical application has been greatly limited due to drug resistance and organ toxicity.

The incidence of drug‐induced hepatotoxicity and nephrotoxicity has been increasing with the ever‐increasing number of drugs and with easy availability of over the counter medications, with hepatotoxicity being important cause of morbidity and mortality and the most common reason for new drugs withdrawal.^4^ Most drug‐induced nephrotoxicities exert toxic effects by one or more common pathological mechanisms, including altered intraglomerular hemodynamics, tubular cell toxicity, inflammation, crystal nephropathy, rhabdomyolysis, and thrombotic microangiopathy.^5^ Redressive measures/interventions are needed to reduce vital organ toxicities posed by doxorubicin, 5‐FU, and other clinically useful chemotherapeutic agents that are normally used at relatively high doses and for an appreciable period of time. Developing new drugs or discovering the other clinical potentials of existing drugs will help in this regard.

Prednisolone (phospholipase A_2_ inhibitor) has been demonstrated to have anti‐inflammatory property, and ameliorate symptoms and improve biochemical and histologic abnormalities in many types of liver diseases, including autoimmune hepatitis, cirrhosis patients with septic shock, and liver transplantation.^6^, ^7^, ^8^, ^9^ Calcium ions (Ca^2+^) are major regulators of vital cellular functions and interference with Ca^2+^ homeostasis contributes to cell injury and death in a number of pathological conditions.^10^, ^11^ Diltiazem is a calcium channel blocker and calcium antagonism/blockade has been exploited in the management of cell injury.^12^

Carvedilol is a third‐generation nonselective beta‐blocker with vasodilatory property due to alpha 1 blockade.^13^ It has also been reported to possess antioxidant property in terms of free radical scavenging and inhibition of lipid peroxidation.^14^, ^15^

This study aimed to determine the protective effect of carvedilol alone and coadministered with diltiazem and prednisolone against doxorubicin and 5‐FU‐induced liver and kidney toxicities.

## 2 MATERIALS AND METHODS

### 2.1 Drugs and chemicals

The following drugs and chemicals were used in this study: Doxorubicin (Get Well Pharmaceuticals, Gurgaon, India), 5‐Fluorouracil (Celon Laboratories Ltd., Gajularamaram, India), Carvedilol (Roche, Mannheim, Germany), Prednisolone (Hovid Berhad, Malaysia), Diltiazem (Sanofi‐Aventis S.p.A., Milan, Italy), and Formalin (Unique Pharmaceuticals, Sango‐Ota, Nigeria).

### 2.2 Animals

Seventy female Wistar rats weighing 150‐200 g were obtained from the Laboratory Animal Centre of the College of Medicine, University of Lagos, Lagos, Nigeria. The animals were housed at 25°C with 12 hours light/dark cycle, allowed to acclimatize for 14 days before commencement of the experiment, and had free access to standard feed (Livestock Feeds Plc., Lagos, Nigeria) and water. The experimental protocol was in conformity with the guidelines of the United States National Academy of Sciences Guide for the Care and Use of Laboratory Animals.^16^

### 2.3 Treatment

Seventy female rats were randomly allotted into 10 groups of 7 animals each and treated as follows:

Group 1 (Control): Normal saline (10 mL/kg).
Group 2: Normal saline (10 mL/kg) and doxorubicin alone (40 mg/kg).
Group 3: Gallic acid (200 mg/kg) and doxorubicin (40 mg/kg) (Positive control).
Group 4: Carvedilol (0.075 mg/kg; subclinical dose) and doxorubicin (40 mg/kg).
Group 5: Carvedilol (0.15 mg/kg; clinical dose) and doxorubicin (40 mg/kg).
Group 6: Carvedilol (0.30 mg/kg; supraclinical dose) and doxorubicin (40 mg/kg).
Group 7: Diltiazem (3.43 mg/kg) and doxorubicin (40 mg/kg).
Group 8: Diltiazem (3.43 mg/kg), carvedilol (0.15 mg/kg), and doxorubicin (40 mg/kg).
Group 9: Prednisolone (0.57 mg/kg) and doxorubicin (40 mg/kg).
Group 10: Prednisolone (0.57 mg/kg), carvedilol (0.15 mg/kg), and doxorubicin (40 mg/kg).

Animals in Group 1 were administered normal saline only for 16 days, while others were separately administered normal saline (Group 2); gallic acid (Group 3); carvedilol (Groups 4‐6); diltiazem (Group 7); diltiazem and carvedilol (Group 8); prednisolone (Group 9); and prednisolone and carvedilol (Group 10) at doses stated above for 16 days. On day 14, doxorubicin was administered to the rats in Groups 2‐10, 2 hours after treatment with the other drugs.^17^ The clinical dose of carvedilol was calculated as the average of doses used for indications of carvedilol, the subclinical dose was half of the clinical dose and the supraclinical dose was twice the clinical dose. A day after the end of administration (day 17), blood samples were collected into plain sample bottles for analysis. Rats were sacrificed by cervical dislocation, laparatomized, and the liver and kidneys were harvested for antioxidant indices and histopathological assessments.

In respect of the 5‐FU model, animals in Group 1 were administered normal saline (10 mL/kg) only for 14 days, while others were separately administered normal saline (Group 2); gallic acid (Group 3); carvedilol (Groups 4‐6); diltiazem (Group 7); diltiazem and carvedilol (Group 8); prednisolone (Group 9); and prednisolone and carvedilol (Group 10) at the same doses used for the doxorubicin model for 14 days. 5‐fluorouracil (20 mg/kg, i.p.) was administered to the rats in Groups 2‐10 from days 10 to 14, 2 hours after treatment with the other drugs.^18^ A day after administration stopped (day 15), blood samples were collected into plain sample bottles for analysis. Rats were sacrificed humanely under inhaled diethyl ether anesthesia, laparatomized and the liver and kidneys were harvested for antioxidant indices and histopathological assessments.

### 2.4 Biochemical analysis

Blood sample was collected from each rat at the end of the drug administration period, via retro‐orbital artery bleeding under anesthesia, into plain sample bottles for biochemical analysis. Blood collected into plain bottles was allowed to clot at room temperature and centrifuged to obtain the serum. The sera were analyzed using Randox diagnostic kits (Randox Laboratories Ltd., London, England) to assess aspartate aminotransferase (AST), alanine aminotransferase (ALT), and alkaline phosphatase (ALP) activities, and the determination of concentrations of serum total protein, albumin, urea, creatinine, high‐density lipoprotein (HDL), low‐density lipoprotein (LDL), cholesterol, and triglycerides (TG), according to established protocols.^19^

### 2.5 Antioxidant indices analysis

Malondialdehyde (MDA) was assayed using the method described by Janero and Burghardt.^20^ Assay of catalase (CAT), superoxide dismutase (SOD), glutathione peroxidase (GPx), and reduced glutathione (GSH) was performed according to established procedures.^21^, ^22^

### 2.6 Histopathological analysis

Rats were sacrificed humanely under inhaled diethyl ether anesthesia, laparatomized and the organs (liver and kidneys) of each animal removed and weighed. The method described by Habbu et al 22 was used to process the tissues for histopathological analysis.

### 2.7 Statistical analysis

Data were analyzed by one‐way ANOVA followed by Tukey's multiple comparison test using GraphPad Prism 5 (GraphPad Software Inc., CA). Results were expressed as mean ± SEM and values were considered significant at *P* < .05.

## 3 RESULTS

### 3.1 Biochemical parameters (doxorubicin model)

Administration of doxorubicin caused a significant (*P* < .05) increase in the levels of AST, ALT, and ALP, as well as a significant (*P* < .05) decrease in albumin relative to the animals administered normal saline alone. In the presence of doxorubicin, administration of carvedilol (all doses) significantly (*P* < .05) decreased AST, ALT, and ALP compared with rats administered doxorubicin alone. Coadministration of diltiazem and carvedilol (in the presence of doxorubicin) caused a significant (*P* < .05) decrease in ALT and AST enzymes compared with rats administered doxorubicin alone. In the presence of doxorubicin, administration of prednisolone and carvedilol resulted in a significant (*P* < .05) decrease in only ALT enzyme (Table 1).

**Table 1:** Effect of carvedilol alone and coadministered with prednisolone and diltiazem (in the presence of doxorubicin) on liver enzymes and albumin

Group	AST (UL^−1^)	ALT (UL^−1^)	ALP (UL^−1^)	ALB (mgL^−1^)
I	99.97 ± 16.52	89.32 ± 19.98	103.45 ± 13.15	30.76 ± 1.93
II	499.50 ± 153.75^a^	312.21 ± 95.51^a^	184.48 ± 31.20^a^	22.96 ± 2.73^a^
III	163.08 ± 40.12^b^	171.77 ± 43.14^b^	91.72 ± 15.73	32.52 ± 1.37
IV	219.20 ± 31.34^b^	111.38 ± 43.91^b^	79.14 ± 20.63	30.69 ± 0.78
V	182.90 ± 23.52^b^	80.52 ± 22.26^b^	162.00 ± 38.82^c^^,^ a	34.86 ± 2.75
VI	258.00 ± 54.75^b^	238.18 ± 70.15^b^	126.76 ± 20.45	37.50 ± 2.16
VII	366.03 ± 62.25	148.28 ± 40.96^b^	108.48 ± 17.54	27.86 ± 3.0
VIII	258.50 ± 23.02^b^^,^ a	160.48 ± 41.30^b^	79.56 ± 4.43	29.75 ± 2.91
IX	348.55 ± 167.11	254. 76 ± 84.91^a^^,^ b	100.82 ± 22.29	25.80 ± 2.52
X	240.07 ± 32.14	270.92 ± 51.26^a^^,^ b	110.20 ± 27.62	32.48 ± 3.40

Group I: Normal saline 10 mL/kg; Group II: Doxorubicin 40 mg/kg; Group III: Gallic acid 200 mg/kg + Doxorubicin 40 mg/kg; Groups IV,V,VI: Carvedilol 0.075 mg/kg, 0.15 mg/kg, 0.30 mg/kg + Doxorubicin 40 mg/kg; Group VII: Diltiazem 3.43 mg/kg + Doxorubicin 40 mg/kg; Group VIII: Diltiazem 3.43 mg/kg + Carvedilol 0.15 mg/kg + Doxorubicin 40 mg/kg; Group IX: Prednisolone 0.57 mg/kg + Doxorubicin 40 mg/kg, Group X: Prednisolone 0.57 mg/kg + Carvedilol 0.15 mg/kg + Doxorubicin 40 mg/kg.

Result expressed as mean ± SEM.

a *P* < .05 vs control group.

b *P *< .05 vs doxorubicin group.

c *P *< .05 vs gallic acid group.

A significant (*P* < .05) increase in creatinine level in animals administered doxorubicin relative to those that received normal saline was observed (Table 2). Administration of carvedilol (all doses), in the presence of doxorubicin, significantly (*P* < .05) decreased creatinine level compared with animals administered doxorubicin alone. In the presence of doxorubicin, coadministration of diltiazem and carvedilol, as well as prednisolone and carvedilol also significantly (*P* < .05) decreased creatinine level relative to rats administered doxorubicin alone (Table 2).

**Table 2:** Effect of carvedilol alone and coadministered with prednisolone and diltiazem (in the presence of doxorubicin) on other biochemical parameters

Group	TP (gL^−1^)	LDH (UL^−1^)	Urea (mmolL^−1^)	Creatinine (μmolL^−1^)
I	72.73 ± 2.37	0.31 ± 0.06	10.02 ± 1.15	41.64 ± 4.26
II	67.48 ± 2.02	0.36 ± 0.04	11.70 ± 2.39	105.77 ± 11.25^a^
III	69.12 ± 3.15	1.30 ± 0.43	11.74 ± 1.74	98.08 ± 6.33^b^
IV	73.01 ± 5.20	0.71 ± 0.28	14.64 ± 1.64	58.65 ± 6.42^b^^,^ c
V	79.57 ± 5.03	0.63 ± 0.13	8.74 ± 0.28	48.76 ± 2.46^b^^,^ c
VI	76.01 ± 6.40	0.55 ± 0.12	8.72 ± 1.12	49.20 ± 5.15^b^^,^ c
VII	62.18 ± 4.44	0.71 ± 0.14	15.60 ± 3.74	48.96 ± 3.63^b^^,^ c
VIII	62.16 ± 4.49	0.96 ± 0.31	12.90 ± 4.25	46.54 ± 4.09^b^^,^ c
IX	61.46 ± 1.29	0.48 ± 0.09	13.86 ± 2.49	92.55 ± 14.02
X	61.52 ± 8.92	0.47 ± 0.10	13.95 ± 2.46	78.22 ± 10.29^b^

Group I: Normal saline 10 mL/kg; Group II: Doxorubicin 40 mg/kg; Group III: Gallic acid 200 mg/kg + Doxorubicin 40 mg/kg; Groups IV,V,VI: Carvedilol 0.075 mg/kg, 0.15 mg/kg, 0.30 mg/kg + Doxorubicin 40 mg/kg; Group VII: Diltiazem 3.43 mg/kg + Doxorubicin 40 mg/kg; Group VIII: Diltiazem 3.43 mg/kg + Carvedilol 0.15 mg/kg + Doxorubicin 40 mg/kg; Group IX: Prednisolone 0.57 mg/kg + Doxorubicin 40 mg/kg, Group X: Prednisolone 0.57 mg/kg + Carvedilol 0.15 mg/kg + Doxorubicin 40 mg/kg.

Result expressed as mean ± SEM.

a *P* < .05 vs control group.

b *P *< .05 vs doxorubicin group.

c *P *< .05 vs gallic acid group.

### 3.2 Antioxidant indices (doxorubicin model)

In respect of the liver, Table 3 shows significant (*P* < .05) decreases in the levels of CAT, SOD, GSH, and GPx, and significant (*P* < .05) increase in MDA level in rats administered doxorubicin alone compared with the animals administered normal saline (control). Carvedilol (0.43 mg/kg) significantly (*P* < .05) reduced the level of MDA relative to the doxorubicin alone administered rats. In the presence of doxorubicin, coadministration of carvedilol and diltiazem significantly (*P* < .05) increased the levels of SOD and CAT, with a significant (*P* < .05) decrease in MDA level relative to the toxicant group. Carvedilol combined with prednisolone (in the presence of the toxicant) caused significant (*P* < .05) increases in CAT and GSH, and a significant (*P* < .05) decrease in MDA compared with the animals administered doxorubicin (toxicant) alone.

**Table 3:** Effect of carvedilol alone and coadministered with prednisolone and diltiazem (in the presence of doxorubicin) on antioxidant indices in the liver

Group	CAT (Umg^−1^)	SOD (Umg^−1^)	GSH (mUmg^−1^)	GP_X_ (mUmg^−1^)	MDA (nmolg^−1^)
I	27.46 ± 2.87	8.58 ± 0.57	0.88 ± 0.15	323.55 ± 27.52	7.61 ± 0.85
II	12.71 ± 1.18^a^	4.77 ± 0.73^a^	0.23 ± 0.32^a^	112.96 ± 22.66^a^	17.49 ± 1.16^a^
III	33.45 ± 4.30	9.16 ± 0.63^b^	1.09 ± 0.63^b^	112.22 ± 5.49^a^^,^ b	5.67 ± 0.87^b^
IV	12.48 ± 1.92^c^	6.57 ± 2.15^c^	0.65 ± 0.08	112.73 ± 1.19^a^	17.47 ± 2.59^a^
V	20.11 ± 4.75^c^	4.98 ± 0.54^a^^,^ c	0.48 ± 0.06	253.04 ± 16.32^c^	10.06 ± 1.38^b^
VI	21.35 ± 7.96^b^^,^ c	9.68 ± 0.50^b^	0.23 ± 0.06^a^^,^ c	116.50 ± 1.24^a^	16.25 ± 1.75^a^
VII	24.79 ± 1.97^b^^,^ c	5.00 ± 0.71^a^	0.39 ± 0.14^c^	257.03 ± 17.93^b^^,^ c	5.85 ± 1.28^b^
VIII	24.35 ± 1.45^b^^,^ c	9.76 ± 0.66^b^^,^ c	0.28 ± 0.04^a^^,^ c	109.65 ± 1.89 a ^,^ c	5.80 ± 1.42^b^
IX	12.47 ± 1.05	7.77 ± 1.00^b^	1.53 ± 0.33^a^^,^ b	278.89 ± 63.72	5.63 ± 2.27^b^
X	21.44 ± 0.82^b^^,^ c	4.46 ± 0.00^a^,^c^	1.46 ± 0.48^b^	178.35 ± 36.52^a^	6.22 ± 3.06^b^

Group I: Normal saline 10 mL/kg; Group II: Doxorubicin 40 mg/kg; Group III: Gallic acid 200 mg/kg + Doxorubicin 40 mg/kg; Groups IV,V,VI: Carvedilol 0.075 mg/kg, 0.15 mg/kg, 0.30 mg/kg + Doxorubicin 40 mg/kg; Group VII: Diltiazem 3.43 mg/kg + Doxorubicin 40 mg/kg; Group VIII: Diltiazem 3.43 mg/kg + Carvedilol 0.15 mg/kg + Doxorubicin 40 mg/kg; Group IX: Prednisolone 0.57 mg/kg + Doxorubicin 40 mg/kg, Group X: Prednisolone 0.57 mg/kg + Carvedilol 0.15 mg/kg + Doxorubicin 40 mg/kg.

Result expressed as mean ± SEM.

a *P* < .05 vs control group.

b *P *< .05 vs doxorubicin group.

c *P *< .05 vs gallic acid group.

In respect of the kidneys, results in Table 4 shows significant (*P* < .05) decreases in the levels of CAT, SOD, and GPx, and a significant (*P* < .05) increase in MDA level following administration of doxorubicin compared with animals administered normal saline (control). Administration of carvedilol in the presence of doxorubicin caused significant (*P* < .05) increases in SOD, CAT, and GPx levels and a significant (*P* < .05) decrease in MDA relative to animals administered doxorubicin alone. In the presence of doxorubicin, coadministration of carvedilol and diltiazem significantly (*P* < .05) increased the levels of SOD, CAT, and GPx, with a significant (*P* < .05) decrease in MDA level relative to the toxicant group. Carvedilol combined with prednisolone (in the presence of the toxicant) caused significant (*P* < .05) increases in the levels of CAT, SOD, and GPx, but insignificant (*P* > .05) decrease in MDA level compared with the rats administered doxorubicin.

**Table 4:** Effect of carvedilol alone and coadministered with prednisolone and diltiazem (in the presence of doxorubicin) on antioxidant indices in the kidney

Group	CAT (Umg^−1^)	SOD (Umg^−1^)	GSH (mUmg^−1^)	GPx (mUmg^−1^)	MDA (nmolg^−1^)
I	23.83 ± 2.47	7.20 ± 0.74	0.43 ± 0.09	193.27 ± 25.53	8.55 ± 0.67
II	10.55 ± 1.80^a^	4.04 ± 0.87^a^	0.21 ± 0.06	64.55 ± 13.5^a^	14.30 ± 1.66^a^
III	26.39 ± 2.72^b^	6.90 ± 0.61^b^	0.28 ± 0.05	146.12 ± 30.56^b^	7.38 ± 0.31^b^
IV	20.24 ± 1.18^b^	6.28 ± 0.29	0.33 ± 0.07	140.60 ± 25.65^b^	13.04 ± 1.54^b^
V	27.50 ± 3.46^b^	8.07 ± 0.88^b^	0.38 ± 0.04	197.93 ± 25.95^b^	7.56 ± 0.80^b^
VI	26.43 ± 3.46^b^	8.28 ± 0.86^b^	0.34 ± 0.06	193.84 ± 25.71^b^	7.57 ± 0.85^b^
VII	24.13 ± 2.75^b^	7.30 ± 1.89^b^	0.65 ± 0.18^b^	100.91 ± 1.26^a^	6.75 ± 0.48^b^
VIII	28.37 ± 3.90^b^	8.31 ± 1.13^b^	0.42 ± 0.06	200.08 ± 33.06^b^	7.96 ± 0.80^b^
IX	26.82 ± 3.86^b^	9.34 ± 0.96^b^	0.30 ± 0.10	110.43 ± 4.04^a^	8.28 ± 0.84^b^
X	24.66 ± 2.28^b^	7.89 ± 0.83^b^	0.47 ± 0.11	190.52 ± 27.44^a^^,^ b	8.98 ± 1.82

Group I: Normal saline 10 mL/kg; Group II: Doxorubicin 40 mg/kg; Group III: Gallic acid 200 mg/kg + Doxorubicin 40 mg/kg; Groups IV,V,VI: Carvedilol 0.075 mg/kg, 0.15 mg/kg, 0.30 mg/kg + Doxorubicin 40 mg/kg; Group VII: Diltiazem 3.43 mg/kg + Doxorubicin 40 mg/kg; Group VIII: Diltiazem 3.43 mg/kg + Carvedilol 0.15 mg/kg + Doxorubicin 40 mg/kg; Group IX: Prednisolone 0.57 mg/kg + Doxorubicin 40 mg/kg, Group X: Prednisolone 0.57 mg/kg + Carvedilol 0.15 mg/kg + Doxorubicin 40 mg/kg.

Result expressed as mean ± SEM.

a *P* < .05 vs control group.

b *P *< .05 vs doxorubicin group.

### 3.3 Biochemical parameters (5‐FU model)

Results showed that administration of 5‐FU significantly (*P* < 0.05) increased AST level compared with normal saline (control) (Table 5). Administration of carvedilol alone and coadministered with diltiazem or prednisolone significantly (*P* < .05) decreased AST level relative to rats that received 5‐FU alone.

**Table 5:** Effect of carvedilol alone and coadministered with prednisolone and diltiazem (in the presence of 5‐FU) on liver enzymes and albumin

Group	AST (UL^−1^)	ALT (UL^−1^)	ALP (UL^−1^)	ALB (gL^−1^)
I	110.04 ± 9.45	111.60 ± 26.30	91.30 ± 25.30	28.30 ± 2.50
II	481.48 ± 126.38^a^	189.30 ± 68.30	105.40 ± 32.30	21.42 ± 2.80
III	202.54 ± 127.92^b^	110.30 ± 24.60	111.70 ± 17.60	25.50 ± 2.10
IV	275.10 ± 65.18^b^	99.20 ± 10.40	110.40 ± 29.00	31.90 ± 2.70
V	243.02 ± 55.01^b^	151.80 ± 35.30	102.30 ± 31.50	24.40 ± 5.20
VI	225.18 ± 45.32^b^	110.60 ± 6.30	110.50 ± 6.60	33.80 ± 0.90
VII	222.12 ± 47.07^b^	155.80 ± 27.80	117.20 ± 25.60	36.10 ± 5.80^b^
VIII	216.68 ± 56.76^b^	156.30 ± 16.60	114.20 ± 23.50	53.20 ± 12.80^a^^,^ b
IX	294.06 ± 82.68	147.20 ± 23.60	130.96 ± 15.50	40.30 ± 1.60^b^^,^ c
X	277.06 ± 87.84^b^	135.50 ± 27.30	128.80 ± 16.00	46.90 ± 3.60^a^^,^ b ^,^ c

Group I: Normal saline 10 mL/kg; Group II: Doxorubicin 40 mg/kg; Group III: Gallic acid 200 mg/kg + Doxorubicin 40 mg/kg; Groups IV,V,VI: Carvedilol 0.075 mg/kg, 0.15 mg/kg, 0.30 mg/kg + Doxorubicin 40 mg/kg; Group VII: Diltiazem 3.43 mg/kg + Doxorubicin 40 mg/kg; Group VIII: Diltiazem 3.43 mg/kg + Carvedilol 0.15 mg/kg + Doxorubicin 40 mg/kg; Group IX: Prednisolone 0.57 mg/kg + Doxorubicin 40 mg/kg, Group X: Prednisolone 0.57 mg/kg + Carvedilol 0.15 mg/kg + Doxorubicin 40 mg/kg.

Result expressed as mean ± SEM.

a *P* < .05 vs control group.

b *P *< .05 vs doxorubicin group.

c *P *< .05 vs gallic acid group.

As shown in Table 6, a significant (*P* < .05) increase in creatinine level was observed in rats administered 5‐FU compared with the control group. In the presence of 5‐FU, administration of carvedilol alone (all doses) and coadministered with diltiazem significantly (*P* < .05) reduced the creatinine level compared with the rats that received 5‐FU only.

**Table 6:** Effect of carvedilol alone and coadministered with prednisolone and diltiazem (in the presence of 5‐FU) on other biochemical parameters

Group	TP (gL^−1^)	LDH (UL^−1^)	Urea (mmolL^−1^)	Creatinine (μmolL^−1^)
I	57.80 ± 6.90	51.70 ± 12.80	8.20 ± 0.90	39.90 ± 3.00
II	52.40 ± 3.10	72.60 ± 32.90	13.10 ± 2.70	73.60 ± 14.20^a^
III	58.60 ± 3.10	93.80 ± 55.90	14.80 ± 3.00	57.40 ± 13.40
IV	56.90 ± 13.10	42.90 ± 15.50	18.00 ± 11.40	43.00 ± 4.50^a^^,^ b
V	65.80 ± 9.60	51.20 ± 14.20	9.60 ± 0.70	37.50 ± 4.60^b^
VI	57.40 ± 14.50	40.90 ± 14.20	8.00 ± 0.70	40.60 ± 4.50^b^
VII	59.70 ± 1.90	136.40 ± 16.90^a^	10.60 ± 2.90	35.60 ± 9.10^b^
VIII	56.12 ± 3.80	125.50 ± 29.30^a^	10.40 ± 2.60	35.80 ± 8.20^b^
IX	55.50 ± 4.51	109.80 ± 11.20	15.70 ± 3.90	47.50 ± 5.30^b^
X	65.70 ± 5.30	104.30 ± 6.60	12.60 ± 3.03	44.90 ± 5.40^b^

Group I: Normal saline 10 mL/kg; Group II: Doxorubicin 40 mg/kg; Group III: Gallic acid 200 mg/kg + Doxorubicin 40 mg/kg; Groups IV,V,VI: Carvedilol 0.075 mg/kg, 0.15 mg/kg, 0.30 mg/kg + Doxorubicin 40 mg/kg; Group VII: Diltiazem 3.43 mg/kg + Doxorubicin 40 mg/kg; Group VIII: Diltiazem 3.43 mg/kg + Carvedilol 0.15 mg/kg + Doxorubicin 40 mg/kg; Group IX: Prednisolone 0.57 mg/kg + Doxorubicin 40 mg/kg, Group X: Prednisolone 0.57 mg/kg + Carvedilol 0.15 mg/kg + Doxorubicin 40 mg/kg.

Result expressed as mean ± SEM.

a *P* < .05 vs control group.

b *P *< .05 vs doxorubicin group.

### 3.4 Antioxidant indices (5‐FU model)

In respect of the liver, significant (*P* < .05) decreases in CAT, SOD, GSH, and GPx levels and a significant (*P* < .05) increase in MDA level were observed in rats administered 5‐FU relative to the normal saline (control) group (Table 7). Carvedilol (0.075 mg/kg) coadministered with 5‐FU produced a significant (*P* < .05) increase in the level of CAT and significant (*P* < .05) decrease in MDA level compared with rats administered 5‐FU alone. Coadministration of carvedilol and prednisolone (in the presence of 5‐FU) caused significant (*P* < .05) increases in the levels of CAT, SOD, and GPx, and a significant (*P* < .05) decrease in MDA level compared with rats that received 5‐FU alone.

**Table 7:** Effect of carvedilol alone and coadministered with prednisolone and diltiazem (in the presence of 5‐FU) on antioxidant indices in the liver

Group	CAT (Umg^−1^)	SOD (Umg^−1^)	GSH (mUmg^−1^)	GP_X_ (mUmg^−1^)	MDA (nmolg^−1^)
I	39.09 ± 4.19	5.51 ± 0.59	0.35 ± 0.04	180.73 ± 14.29	6.00 ± 1.08
II	15.05 ± 1.10^a^	3.73 ± 0.60^a^	0.19 ± 0.06^a^	8.47 ± 4.77^a^	13.80 ± 2.37^a^
III	54.07 ± 3.57^a^^,^ b	7.39 ± 0.97^a^^,^ b	0.20 ± 0.03^a^	90.60 ± 4.55^a^	4.41 ± 0.73^b^
IV	22.95 ± 5.76^a^^,^ b ^,^ c	3.73 ± 0.25^a^^,^ c	0.18 ± 0.02^a^	128.84 ± 14.20^a^	87.16 ± 1.27^b^
V	24.27 ± 5.80^a^^,^ c	4.85 ± 0.36^c^	0.20 ± 0.07^a^	89.13 ± 3.40^a^	7.06 ± 1.28^b^
VI	18.56 ± 4.02^a^^,^ c	3.94 ± 0.76^c^	0.20 ± 0.03^a^	126.15 ± 24.86^a^	13.46 ± 0.78^a^^,^ b ^,^ c
VII	30.57 ± 4.64^a^^,^ b ^,^ c	4.01 ± 0.32^c^	0.17 ± 0.02	104.86 ± 2.46^a^	3.98 ± 0.79^b^
VIII	30.32 ± 3.37^c^	4.02 ± 0.36^c^	0.25 ± 0.05	104.51 ± 9.32^a^	3.96 ± 0.76^b^
IX	15.10 ± 0.98^a^^**,**^ c	3.48 ± 0.38^a^	0.37 ± 0.08^b^^,^ c	135.90 ± 28.65^a^^,^ b	8.32 ± 1.93^b^
X	28.95 ± 3.49^b^^,^ c	6.18 ± 0.98^b^^,^ c	0.28 ± 0.06	136.41 ± 21.18^a^^,^ b	6.04 ± 0.38^b^

Group I: Normal saline 10 mL/kg; Group II: Doxorubicin 40 mg/kg; Group III: Gallic acid 200 mg/kg + Doxorubicin 40 mg/kg; Groups IV,V,VI: Carvedilol 0.075 mg/kg, 0.15 mg/kg, 0.30 mg/kg + Doxorubicin 40 mg/kg; Group VII: Diltiazem 3.43 mg/kg + Doxorubicin 40 mg/kg; Group VIII: Diltiazem 3.43 mg/kg + Carvedilol 0.15 mg/kg + Doxorubicin 40 mg/kg; Group IX: Prednisolone 0.57 mg/kg + Doxorubicin 40 mg/kg, Group X: Prednisolone 0.57 mg/kg + Carvedilol 0.15 mg/kg + Doxorubicin 40 mg/kg.

Result expressed as mean ± SEM.

a *P* < .05 vs control group.

b *P *< .05 vs doxorubicin group.

c *P *< .05 vs gallic acid group.

**Table 8:** Effect of carvedilol alone and coadministered with prednisolone and diltiazem (in the presence of 5‐FU) on antioxidant indices in the kidney

Group	CAT (Umg^−1^)	SOD (Umg^−1^)	GSH (mUmg^−1^)	GP_X_ (mUmg^−1^)	MDA (nmolg^−1^)
I	4.22 ± 1.73	6.17 ± 0.94	0.56 ± 0.10	113.63 ± 7.47	7.93 ± 0.30
II	9.28 ± 1.18^a^	4.24 ± 0.42	0.20 ± 0.05^a^	71.65 ± 8.61	13.40 ± 1.80^a^
III	26.47 ± 1.18^b^	10.16 ± 1.57^a^^,^ b	0.44 ± 0.07	84.15 ± 20.51	7.44 ± 0.44^b^
IV	13.84 ± 3.54^a^^,^ b ^,^ c	8.39 ± 1.64^b^	0.46 ± 0.14^b^	82.87 ± 12.84	10.57 ± 1.96^c^
V	23.62 ± 4.28^b^	6.64 ± 0.43^c^	0.55 ± 0.14^b^	110.02 ± 10.65	8.16 ± 0.73^b^
VI	24.14 ± 4.46^b^	6.67 ± 0.54^c^	0.59 ± 0.10^b^	106.24 ± 9.55	10.48 ± 2.44
VII	18.59 ± 2.63^b^^,^ c	6.05 ± 0.74	0.60 ± 0.06^a^	108.65 ± 11.68	8.57 ± 0.68
VIII	18.39 ± 3.45	7.41 ± 0.28^b^^,^ c	0.61 ± 0.10^b^	318.70 ± 205.24^a^^,^ b ^,^ c	7.86 ± 0.52^b^
IX	20.14 ± 0.43	7.26 ± 1.04^b^^,^ c	0.20 ± 0.05^a^	86.33 ± 9.05	6.18 ± 1.12^b^
X	24.84 ± 1.78	6.33 ± 0.87	0.58 ± 0.01^b^	107.31 ± 7.33	8.00 ± 1.17^b^

Group I: Normal saline 10 mL/kg; Group II: Doxorubicin 40 mg/kg; Group III: Gallic acid 200 mg/kg + Doxorubicin 40 mg/kg; Groups IV,V,VI: Carvedilol 0.075 mg/kg, 0.15 mg/kg, 0.30 mg/kg + Doxorubicin 40 mg/kg; Group VII: Diltiazem 3.43 mg/kg + Doxorubicin 40 mg/kg; Group VIII: Diltiazem 3.43 mg/kg + Carvedilol 0.15 mg/kg + Doxorubicin 40 mg/kg; Group IX: Prednisolone 0.57 mg/kg + Doxorubicin 40 mg/kg, Group X: Prednisolone 0.57 mg/kg + Carvedilol 0.15 mg/kg + Doxorubicin 40 mg/kg.

Result expressed as mean ± SEM.

a *P* < .05 vs control group.

b *P *< .05 vs doxorubicin group.

c *P *< .05 vs gallic acid group.

In respect of the kidney, 5‐FU administration significantly (*P* < .05) decreased GSH and increased MDA levels compared with rats administered normal saline (control) (Table 8). Carvedilol (0.075 mg/kg) in the presence of 5‐FU increased CAT, SOD, and GSH levels significantly (*P* < .05). In the presence of 5‐FU, a higher dose of carvedilol (0.15 mg/kg) caused significant (*P* < .05) increases in the levels of CAT and GSH, and a significant (*P* < .05) decrease in MDA level compared with rats that received 5‐FU only. Coadministration of carvedilol and diltiazem significantly (*P* < .05) increased the levels of SOD, GSH, and GPx, and decreased the MDA level significantly (*P* < .05) compared with rats that received 5‐FU only. Carvedilol administered with prednisolone increased GSH and decreased MDA levels significantly (*P* < .05) relative to the 5‐FU only group.

### 3.5 Histopathological analyses

**Figure 1:**
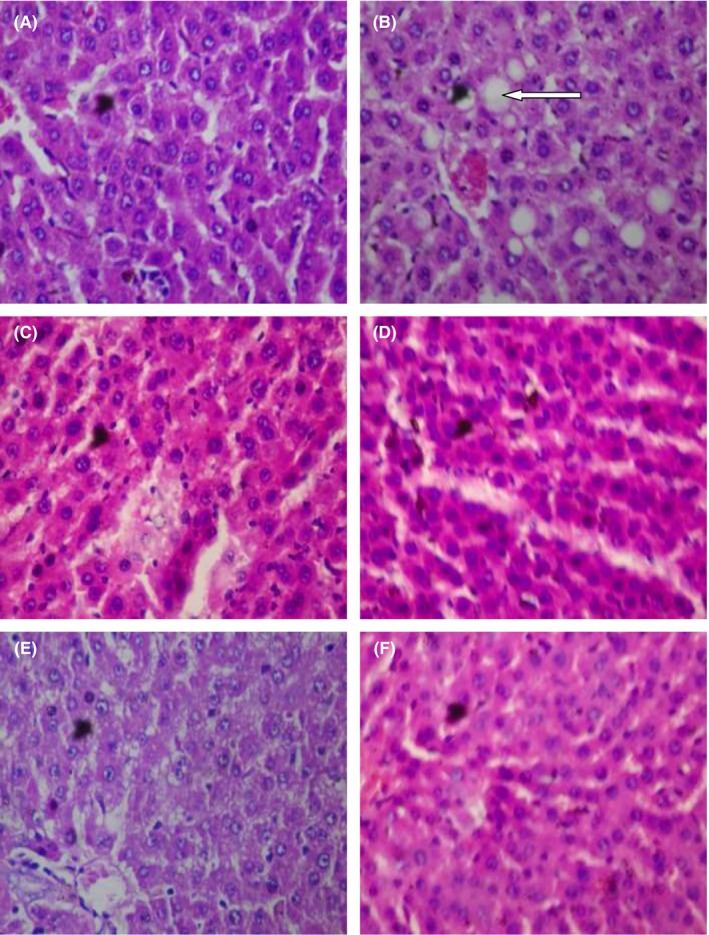
Photomicrographs of liver sections showing effect of carvedilol alone and coadministered with prednisolone and diltiazem (in the presence of doxorubicin) (H&E stain; ×400). (A) Represents control treated with normal saline 10 mL/kg p.o. (showing normal architecture); (B) represents group treated with doxorubicin 40 mg/kg i.p. (arrow showing steatosis), (C) represents group treated with doxorubicin (40 mg/kg i.p.) + gallic acid (200 mg/kg p.o.) (showing normal architecture), (D) represents group treated with doxorubicin (40 mg/kg i.p.) + carvedilol (0.15 mg/kg p.o.) (showing normal architecture), (E) represents group treated with doxorubicin (40 mg/kg i.p.) + diltiazem (3.43 mg/kg p.o.) + carvedilol (0.15 mg/kg p.o.) (showing normal architecture), and (F) represents group treated with doxorubicin (40 mg/kg i.p.) + prednisolone (0.57 mg/kg p.o.) + carvedilol (0.15 mg/kg p.o.) (showing normal architecture)

**Figure 2:**
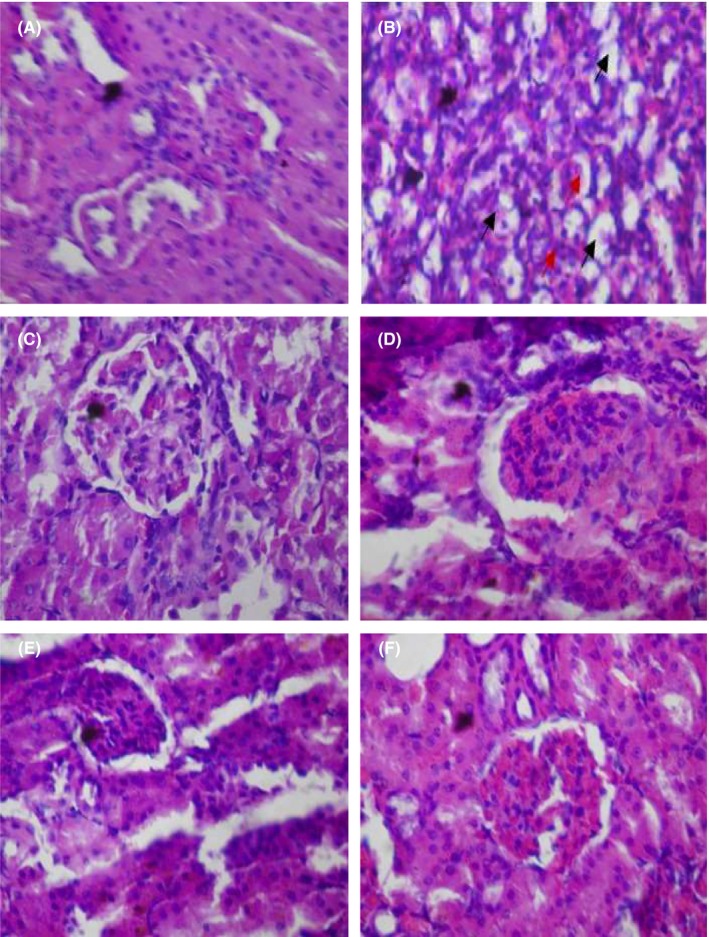
Photomicrographs of kidney sections showing effect of carvedilol alone and coadministered with prednisolone and diltiazem (in the presence of doxorubicin) (H&E stain; ×400). (A) Represents control treated with normal saline 10 mL/kg p.o. (showing normal architecture), (B) represents group treated with doxorubicin 40 mg/kg i.p. (black arrows: cortical necrosis; red arrows: thin glomeruli basement membrane), (C) represents group treated with doxorubicin (40 mg/kg i.p.) + gallic acid (200 mg/kg p.o.) (normal), (D) represents group treated with doxorubicin (40 mg/kg i.p.) + carvedilol (0.15 mg/kg p.o.) (normal), (E) represents group treated with doxorubicin (40 mg/kg i.p.) + diltiazem (3.43 mg/kg p.o.) + carvedilol (0.15 mg/kg p.o.) (normal), and (F) represents group treated with doxorubicin (40 mg/kg i.p.) + prednisolone (0.57 mg/kg p.o.) + carvedilol (0.15 mg/kg p.o.) (normal)

**Figure 3:**
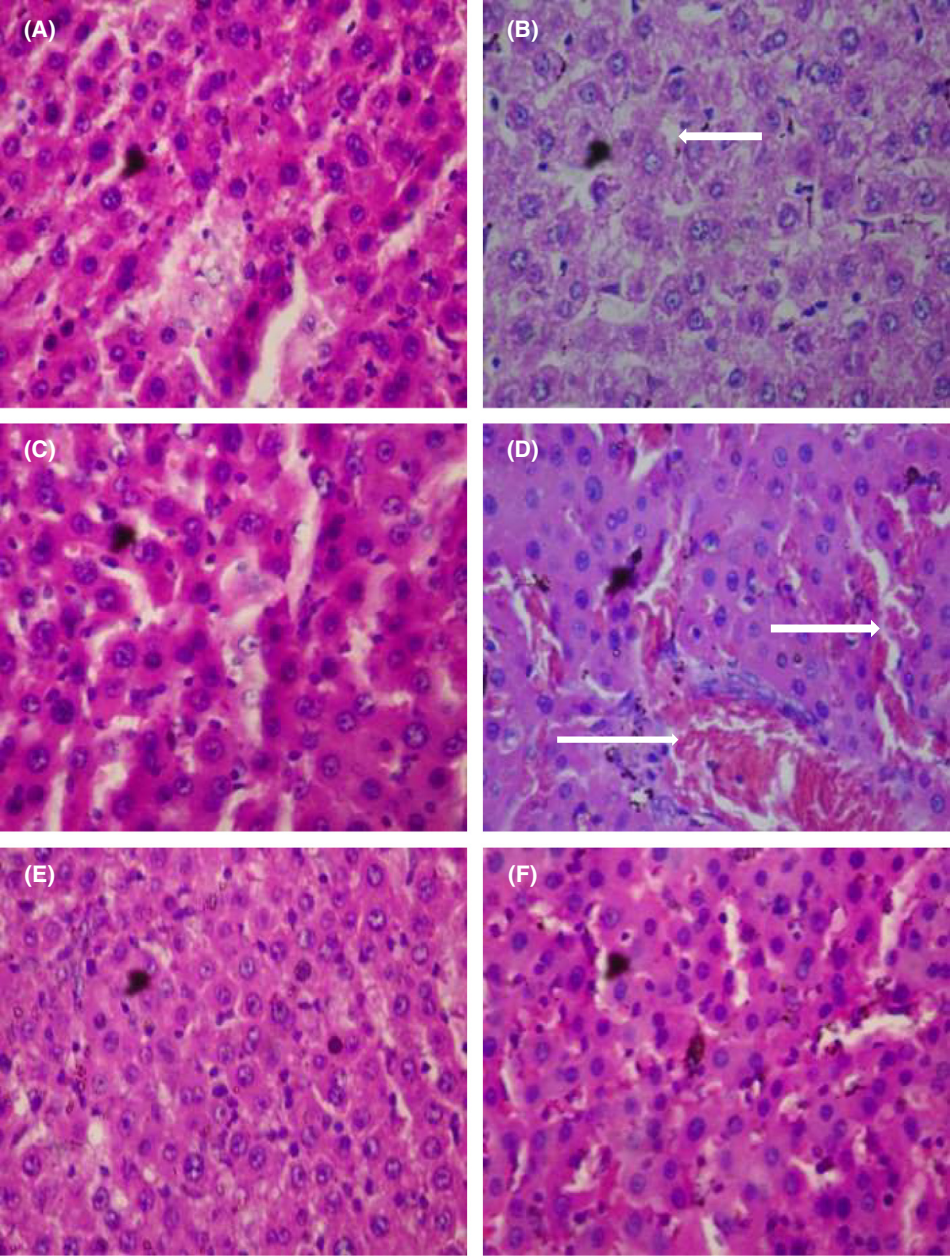
Photomicrographs of liver sections showing effect of carvedilol alone and coadministered with prednisolone and diltiazem (in the presence of 5‐FU) (H&E stain; ×400). (A) Represents control treated with normal saline 10 mL/kg p.o. (showing normal architecture), (B) represents group treated with 5‐FU 20 mg/kg i.p. (arrow showing steatosis), (C) represents group treated with 5‐FU (20 mg/kg i.p.) + gallic acid (200 mg/kg p.o.) (showing normal architecture), (D) represents group treated with 5‐FU (20 mg/kg i.p.) + carvedilol (0.15 mg/kg p.o.) (arrows showing congestion of vascular channels), (E) represents group treated with 5‐FU (20 mg/kg i.p.) + carvedilol (0.15 mg/kg p.o.) + diltiazem (3.43 mg/kg p.o.) (showing normal architecture), and (F) represents group treated with 5‐FU (20 mg/kg i.p.) + carvedilol (0.15 mg/kg p.o.) + prednisolone (0.57 mg/kg p.o.) (showing normal architecture)

**Figure 4:**
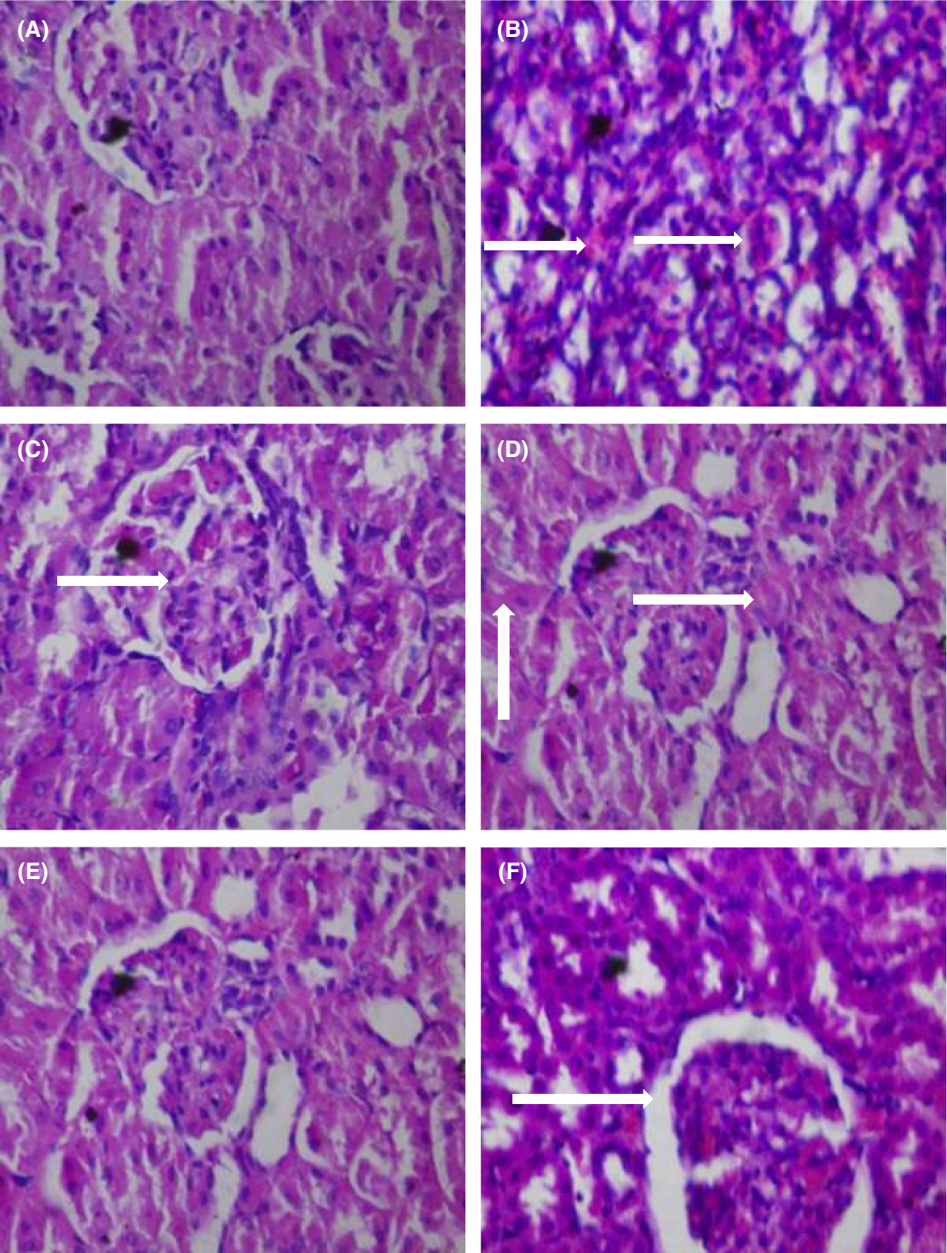
Photomicrographs of kidney sections showing effect of carvedilol alone and coadministered with prednisolone and diltiazem (in the presence of 5‐FU) (H&E stain; ×400). (A) Represents control treated with normal saline 10 mL/kg p.o. (showing normal architecture), (B) represents group treated with 5‐FU 20 mg/kg i.p. (arrows showing local hemorrhage), (C) represents group treated with 5‐FU (20 mg/kg i.p.) + gallic acid (200 mg/kg p.o.) (arrow showing glomerular cell ballooning), (D) represents group treated with 5‐FU (20 mg/kg i.p.) + carvedilol (0.15 mg/kg p.o.) (arrows showing congestion of vascular channels), (E) represents group treated with 5‐FU (20 mg/kg i.p.) + carvedilol (0.15 mg/kg p.o.) + diltiazem (3.43 mg/kg p.o.) (showing normal architecture), and (F) represents group treated with 5‐FU (20 mg/kg i.p.) + carvedilol (0.15 mg/kg p.o.) + prednisolone (0.57 mg/kg p.o.) (arrow showing tubular vacuolation)

Doxorubicin caused steatosis in liver cells relative to the control group which manifested normal architecture. Other treatment groups also showed normal architecture (Figure 1). In respect of the kidney, doxorubicin caused cortical necrosis with thin glomeruli basement membrane relative to the control group with normal architecture. The histoarchitecture of the other treatment groups were normal (Figure 2). 5‐fluorouracil compared with the control group caused steatosis in the liver cells. Presentations in the other treatment groups were normal (Figure 3). Local hemorrhage was observed in the kidney cells of 5‐fluorouracil‐treated rats relative to the control group. Glomerular cell ballooning was observed in the group that received 5‐FU plus gallic acid, while congestion of vascular channels was observed in the group that received 5‐FU plus carvedilol. The group that was given 5‐FU plus carvedilol showed normal architecture, while the group that received 5‐FU plus carvedilol plus prednisolone manifested tubular vacuolation (Figure 4).

## 4 DISCUSSION

The protective role of carvedilol administered alone and with either prednisolone or diltiazem in doxorubicin‐ and 5‐fluorouracil‐induced hepato‐ and nephrotoxicity in female Wistar rats was examined in this study. Liver function tests are a useful diagnostic tool; thus, an elevation in AST and the liver‐specific ALT indicates leakage from injured tissues caused by hepatocellular necrosis,^23^ while increase in ALP level is due to overproduction and release in blood following hepatobiliary injury and cholestasis.^24^ In this study, significant increases in serum AST, ALT, and ALP levels compared with control were observed after administration of doxorubicin and 5‐FU. This clearly indicates the toxic effects of these agents in the liver. Rajesh et al^25^ reported similar findings in an earlier study. Decrease in the level of albumin observed following administration of doxorubicin and 5‐FU is indicative of the consequences of decreased protein synthesis via hepatic dysfunction^26^ or increased protein loss through the gut or the kidney.^27^ Gallic acid, which has known antioxidant and anti‐inflammatory properties, served the purpose of being a standard agent in this study.

Administration of carvedilol in the presence of doxorubicin caused a significant decrease in ALT but the reduction in the level of this liver‐specific enzyme was nonsignificant in the 5‐FU model. Compared with the animals administered doxorubicin alone, coadministration of carvedilol and prednisolone (in the presence of doxorubicin) resulted in a significant decrease in ALT, while AST and ALT were significantly reduced when carvedilol and diltiazem were coadministered. A comparison of coadministration of carvedilol with diltiazem and carvedilol with prednisolone (all in the presence of 5‐FU) revealed a similar situation as the levels of AST and albumin were significantly decreased and increased, respectively. While the inability of these 2 drug combinations (carvedilol with diltiazem and carvedilol with prednisolone) to significantly reduce ALT (in the presence of 5‐FU) is a pointer to their relatively weak protective effect compared with that involving doxorubicin, it is clear that the combinations used in this study did not necessarily confer any advantage as against using a single drug. Although the presence of diltiazem did not add any advantage as against using carvedilol alone in this study, the ability of diltiazem to protect or reduce injury caused by toxicants had earlier been reported by Bojanić et al.^28^

This study revealed that doxorubicin and 5‐FU, known nephrotoxicants,^17^, ^29^ caused a deterioration of renal function as observed by a significant increase in creatinine, with a nonsignificant increase in urea, compared with the control group. Urea and creatinine taken together gives very accurate estimation of kidney function; however, creatinine is a more accurate predictor of kidney damage or injury than urea, though both the liver and kidney must be functioning properly for the body to maintain a normal level of urea in the blood.^30^

In this study, carvedilol administered in the presence of doxorubicin and 5‐FU significantly decreased creatinine level compared with administration of the toxicants alone. This is a pointer to the nephroprotective effect of carvedilol, an observation supported by the findings of Wong et al^31^ and Pathak et al.^32^ Coadministration of carvedilol and diltiazem in the presence of the 2 toxicants resulted in a significant decrease in creatinine level compared with administration of the toxicants alone. Urea decreased as well but nonsignificantly. This indicates the renoprotection offered by this drug combination. Replacing diltiazem with prednisolone similarly decreased creatinine level in a significant manner following exposure to 5‐FU, but nonsignificantly in the presence of doxorubicin. Comparing the result for the single drug administration (carvedilol in the presence of the toxicants) with that of the combination of drugs does not show a clear advantage of one over the other.

Oxidative stress occurs when there is an imbalance between pro‐oxidants and antioxidants in favor of the former, which is very harmful to cells.^33^ Free radicals that are produced due to oxidative stress engage and overwhelm antioxidant enzymes, resulting in the depletion of the antioxidant defenses and induction of lipid peroxidation evident in elevation of MDA level.^34^, ^35^ This contributes to the initiation and progression of hepatic damage in a variety of liver disorders.^36^ In order to maintain the stability in living organisms, it is necessary to maintain balance between the oxidative and antioxidant defense.^37^

An elevation in MDA level usually occurs with a decrease in endogenous antioxidants (SOD, CAT, GPx, and GSH) in the presence of oxidative stress.^38^ This correlates with the result from this study, where doxorubicin caused significant decreases in hepatic SOD, CAT, GPx, and GSH, and significantly increased MDA compared with the control group. This clearly demonstrates the ability of doxorubicin to cause oxidative stress resulting in injury to the liver cells. The supratherapeutic dose of carvedilol in the presence of doxorubicin significantly increased the levels of hepatic SOD and CAT, and decreased MDA level compared with doxorubicin administration alone. This result, indicative of the protective effect of carvedilol, is similar to that reported by Ronsein et al^39^ who studied the cytoprotective effects of carvedilol against oxygen free radical generation in the rat liver. The authors concluded that the observed activity of carvedilol was due to its inherent antioxidant activity. Another study showed that carvedilol prevented mitochondrial dysfunction and renal cell death through protection against oxidative stress.^40^ Coadministration of carvedilol with diltiazem and carvedilol with prednisolone resulted in a significant decrease in hepatic MDA level and a significant increase in CAT level in both groups. Comparing these drug combinations with carvedilol administered alone did not show any advantage of one over the other.

Doxorubicin significantly reduced the levels of renal SOD, CAT, GSH, and GPx, while elevating MDA level compared with the control group, which indicates nephrotoxicity. Carvedilol alone or when combined with diltiazem or prednisolone significantly increased renal levels of SOD, CAT, and GPx, and significantly reduced MDA level compared with doxorubicin administration alone. This is suggestive of nephroprotective effect and these results are in agreement with earlier studies conducted on doxorubicin‐induced hepatotoxicity and nephrotoxicity.^41^, ^42^ A comparison between the groups administered carvedilol alone, carvedilol with diltiazem and carvedilol with prednisolone did not show any clear advantage of one over the other. A similar result was obtained with the 5‐FU model for the liver and kidney.

The histopathology photomicrographs for the liver of animals administered doxorubicin and 5‐FU alone indicated the presence of fatty deposits (steatosis), thus confirming the results of the biochemical and antioxidant indices analyses in which case liver enzymes and MDA were elevated following doxorubicin and 5‐FU‐induced toxicities, respectively. The result in our present study is similar to that of El‐Sayyad et al^43^ who reported that light microscopic observations revealed hepatotoxicity caused by doxorubicin and 5‐FU treatment.

Coadministration of carvedilol and doxorubicin/5‐FU did not alter the architecture of the liver, which is an indication of the hepatoprotective effect of carvedilol. In the presence of doxorubicin and 5‐FU, coadministration of carvedilol with diltiazem, and prednisolone with carvedilol did not affect the liver's architecture in both models. This observation correlates with the results of biochemical and antioxidant indices analyses in which case coadministration of carvedilol with diltiazem/prednisolone protected the liver against the injurious effects of doxorubicin and 5‐FU.

Photomicrographs from the kidney samples showed cortical necrosis and thin glomeruli basement membrane caused by doxorubicin and signs of local hemorrhage due to 5‐FU. This confirms the nephrotoxic effects of doxorubicin and 5‐FU observed from the results of the biochemical analysis in which case deterioration of renal function, indicated by increase in creatinine and urea, was observed. In the presence of 5‐FU, the nephroprotective effect of carvedilol was not evident as congestion of vascular channels was seen. Similarly, coadministration of carvedilol and prednisolone (in the presence of 5‐FU) showed signs of tubular vacuolation. In the doxorubicin model, carvedilol alone and coadministered with diltiazem and prednisolone effectively protected the kidney, as no disruption to the integrity and structure was observed. The observation in the 5‐FU model is similar to that of the results of the biochemical analysis, where carvedilol with prednisolone did not completely protect against 5‐FU intoxication and points to a relatively weak protective effect compared with that involving doxorubicin. The reason for this is the ability of carvedilol to inhibit exogenous nicotinamide adenine dinucleotide dehydrogenase (NADH‐D), the enzyme implicated in doxorubicin‐induced production of reactive oxygen species.^44^ The nephroprotective effect of carvedilol has been reported in hypertensive‐stroke prone rats,^31^ owing to its additional antioxidant activity.^45^ A comparison of the effect of administration of carvedilol alone and coadministered with diltiazem and prednisolone (in the doxorubicin and 5‐FU models) did not show any advantage of one over the other.

## 5 CONCLUSION

Carvedilol administered alone and coadministered with diltiazem and prednisolone reduced the effect of doxorubicin and 5‐fluorouracil‐induced hepatic and renal toxicities due to enhanced in vivo antioxidant activity. The protective effect of the interventions was, however, more prominent in the doxorubicin model compared with the 5‐fluorouracil test. Coadministration of carvedilol with either diltiazem or prednisolone did not show better protection relative to carvedilol alone.

## DISCLOSURE

The authors have no conflict of interest to declare in respect of this study.
